# NF-κB p65 regulates hepatic lipogenesis by promoting nuclear entry of ChREBP in response to a high carbohydrate diet

**DOI:** 10.1016/j.jbc.2021.100714

**Published:** 2021-04-27

**Authors:** P. Vineeth Daniel, Surbhi Dogra, Priya Rawat, Abhinav Choubey, Aiysha Siddiq Khan, Sangam Rajak, Mohan Kamthan, Prosenjit Mondal

**Affiliations:** 1 School of Basic Sciences, Indian Institute of Technology Mandi, Mandi, Himachal Pradesh, India; 2 Department of Biochemistry, School of Chemical and Life Sciences Jamia Hamdard, New Delhi, India; 3 Department of Endocrinology, Sanjay Gandhi Post Graduate Institute of Medical Sciences, Lucknow, India

**Keywords:** ChREBP, sorcin, NF-κB p65, high carbohydrate diet, dyslipidemia, lipid partitioning, ACC, acetyl-CoA carboxylase, AMPK, AMP-activated protein kinase, ChREBP, carbohydrate response element-binding protein, DNL, *de novo* lipogenesis, FAO, fatty acid oxidation, FASN, fatty acid synthase, ICC, immunocytochemistry, NAFLD, nonalcoholic fatty liver disease, NCoRI, nuclear receptor corepressor 1, NF-κB, nuclear factor kappa-light chain enhancer of activated B cells, OCR, oxygen consumption rate, PDTC, pyrrolidine dithiocarbamate, SREBP-1c, sterol regulatory element-binding transcription factor 1

## Abstract

Overconsumption of sucrose and other sugars has been associated with nonalcoholic fatty liver disease (NAFLD). Reports suggest hepatic *de novo* lipogenesis (DNL) as an important contributor to and regulator of carbohydrate-induced hepatic lipid accumulation in NAFLD. The mechanisms responsible for the increase in hepatic DNL due to overconsumption of carbohydrate diet are less than clear; however, literatures suggest high carbohydrate diet to activate the lipogenic transcription factor carbohydrate response element-binding protein (ChREBP), which further transcribes genes involved in DNL. Here, we provide an evidence of an unknown link between nuclear factor kappa-light chain enhancer of activated B cells (NF-κB) activation and increased DNL. Our data indicates high carbohydrate diet to enforce nuclear shuttling of hepatic NF-κB p65 and repress transcript levels of sorcin, a cytosolic interacting partner of ChREBP. Reduced sorcin levels, further prompted ChREBP nuclear translocation, leading to enhanced DNL and intrahepatic lipid accumulation both *in vivo* and *in vitro*. We further report that pharmacological inhibition of NF-κB abrogated high carbohydrate diet–mediated sorcin repression and thereby prevented ChREBP nuclear translocation and this, in turn, attenuated hepatic lipid accumulation both in *in vitro* and *in vivo*. Additionally, sorcin knockdown blunted the lipid-lowering ability of the NF-κB inhibitor *in vitro*. Together, these data suggest a heretofore unknown role for NF-κB in regulating ChREBP nuclear localization and activation, in response to high carbohydrate diet, for further explorations in lines of NAFLD therapeutics.

Nonalcoholic fatty liver disease (NAFLD) is defined as a clinical state of excessive accumulation of lipids within hepatic tissue without any history of alcohol abuse. Higher intake of dietary components like carbohydrates (fructose, sucrose, and glucose) promotes chronic positive energy balance fostering hepatic manifestations like NAFLD (1). One of the hallmarks signaling causing NAFLD is abnormally elevated *de novo* lipogenesis (DNL). NAFLD patients have been reported with enhanced DNL and correspondingly high levels of enzymes required for DNL (2, 3, 4, 5, 6, 7, 8, 9). DNL is a highly dynamic and well-coordinated biochemical process with the involvement of prominent transcriptional regulators like carbohydrate response element-binding protein (ChREBP) and SREBP-1c (sterol regulatory element-binding transcription factor 1) (10).

ChREBP is a master regulator of lipid metabolism present across various organs and majorly regulates hepatic DNL. On its nuclear entry, ChREBP binds to highly conserved carbohydrate response element regions across the promoters of ChREBP responsive target genes coding for key enzymes of DNL-like fatty acid synthase (FASN), acetyl-CoA carboxylase (ACC), and stearoyl-CoA desaturase (11). In a study, genetically obese mice (ob/ob) and as well as human fatty liver biopsy samples were seen to have increased expression of ChREBP and elevated transcripts of lipogenic genes (12). Although a high carbohydrate diet is known to be an etiological parameter related to the development of metabolic diseases, ChREBP seems to be a hub protein that translates the carbohydrate-dependent signaling for glucose disposal as lipids (13, 14). Moreover, few studies have also shown inhibition of ChREBP to ameliorate NAFLD in ob/ob and diet-induced obese mice (12, 15). Activation of ChREBP includes several posttranslational modifications like phosphorylation and acetylation as described in the cited articles (16, 17, 18). Cytoplasmic sequestration of ChREBP with adaptor proteins like 14-3-3β or sorcin stands out to be another promising axis of ChREBP activation (19, 20, 21).

Sorcin (soluble resistance-related calcium-binding protein) is a calcium-binding protein belonging to penta-EF-hand family. It is reported to be an important regulator of calcium homeostasis in organs like the heart and brain by inhibiting (ryanodine receptor) RyR activity and Ca^2+^-induced Ca^2+^ release. A study reports sorcin to interact with RyR, pore-forming α1 subunit of voltage-dependent L-type Ca^2+^ channels (L-type VDCC), sarcoendoplasmic reticulum Ca^2+^ ATPase pumps, to potentially monitor Ca^2+^ dependent intracellular excitation-contraction (22). Sorcin is poorly understood in the context of its regulation and mechanism of tissue-specific functions. An interaction study demonstrates sorcin to interact with ChREBP and thereby regulate glucose sensing and metabolism in pancreatic β cells (21). Adding to the same, our lab recently reported Pb^2+^ exposure to downregulate hepatic sorcin levels and thereby enrich ChREBP transactivation and steatosis in liver (20). As sorcin seemed to be regulating ChREBP-mediated hepatic DNL, we were keen to assess the effect of dietary carbohydrates on hepatic sorcin expression and its crosstalk with ChREBP to the progression of hepatic lipid accumulation.

We observed high carbohydrate diet (30% sucrose for *in vivo* model and 30 mM glucose for *in vitro* model) to lower hepatic sorcin protein levels and thereby set cytosolic ChREBP free for nuclear entry and transcriptional activity. As dietary carbohydrate appears to be a universal causative agent of NAFLD progression, it seemed rationale to investigate the regulation of sorcin by high carbohydrate diet.

The nuclear factor kappa-light chain enhancer of activated B cells (NF-κB) signaling stands pivotal across many complexly coordinated biological processes (23). Pieces of literature broadly present NF-κB activation to be either canonical owing to inflammatory stimuli or noncanonical owing to developmental cues (23). Five cytosolic NF-κB subunits form potentially 15 complexes of homodimers/heterodimers of RelA/p65, cRel, RelB, NFκB1/p52, and NFκB2/p50. These subunits through their conserved Rel Homology Domain interact with the κβ sites of target genes and regulate transcription. Additionally, the p65, cRel, RelB possess a transactivation domain through which their transcriptional regulation could be remodeled (24, 25). We found high carbohydrate diet downregulates hepatic sorcin expression through NFκβ-NCoRI–mediated transcriptional repression.

Herein, using numerous complementary experimental approaches, we show the critical participation of NF-κB in ChREBP’s nuclear entry on exposure to excessive carbohydrate diet and linked fatty liver pathologies. Our findings highlight dietary carbohydrate–induced NF-κB nuclear localization and its significance in regulating hepatic sorcin, which in turn regulates ChREBP. Our studies also indicate PDTC (pyrrolidine dithiocarbamate; [NF-κB inhibitor]) to curtail high carbohydrate diet–induced hepatic lipid accumulation by attenuating excessive carbohydrate–induced ChREBP’s nuclear entry. Taken together, we strongly advocate NF-κB p65 to be a critical hub protein with great therapeutic potential in NAFLD

## Results

### High carbohydrate diet induces ChREBP transactivation *via* sorcin

**Figure 1:**
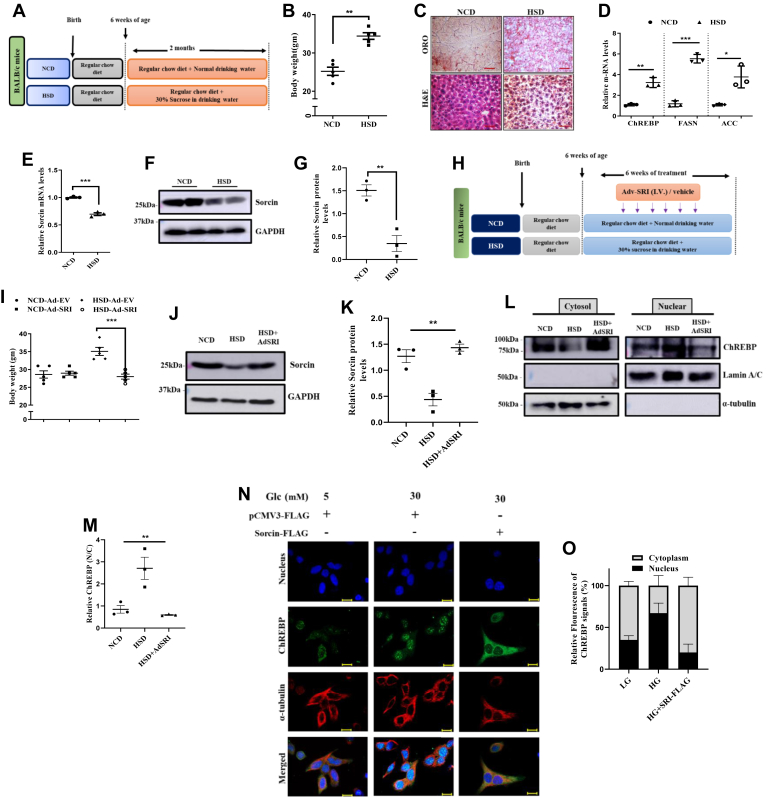
**High carbohydrate diet induces ChREBP transactivation *via* sorcin.***A*, schematic description of the treatment regimens followed in the *in vivo* study. *B*, graphical presentation of body weight gain across the mice within both the groups (NCD, HSD) (mean ± SEM, ∗*p* < 0.05, ∗∗*p* < 0.005). *C*, histological analysis of liver tissue sections through oil red O staining and H&E staining from both NCD, HSD group (Image scale bar is 500 μm). *D*, relative qPCR data of lipogenic genes assessed within the mice liver from both NCD and HSD groups (mean ± SEM, ∗*p* < 0.05, ∗∗*p* < 0.005, ∗∗∗*p* < 0.0005). *E*, transcript levels of sorcin within mice liver isolates, inferring HSD to significantly reduce sorcin levels as compared with NCD (mean ± SEM, ∗∗∗*p* < 0.0005). *F* and *G*, qualitative and quantitative representation of sorcin protein levels within NCD and HSD liver lysates normalized to loading control (mean ± SEM, ∗*p* < 0.05, ∗∗*p* < 0.005). *G*, diagrammatic description of the mice groups used in this strategized study for adenoviral studies. *H*, pictorial representation of the adenoviral treatment regimen used for our *in vivo* study. *I*, graphical presentation of body weight gain across the mice within both the groups (NCD, HSD) (mean ± SEM, ∗*p* < 0.05, ∗∗*p* < 0.005). *J* and *K*, expression abundance of sorcin titers within the mice liver post Adv-SRI (HSD) and Adv-EV injections (NCD, HSD) in the respective groups, as depicted (mean ± SEM, ∗*p* < 0.05, ∗∗*p* < 0.005). *L* and *M*, qualitative and quantitative representation of ChREBP levels in the subcellular fractionated liver lysate of different mice group of adenoviral study. *N* and *O*, representative microscopic images of immunofluorescent tracking of ChREBP within the subcellular regions in presence of different conditions in HepG2 cells and the final quantification of ChREBP signals from the same (Image scale bar is 10 μm). ChREBP, carbohydrate response element-binding protein.

We developed a high sucrose–fed mouse model (Fig. 1*A*) to provide a deeper understanding of endogenous mechanisms-underlying sucrose-induced fatty liver diseases. Mice maintained on regular chow diet (Fig. 1*A*) when exposed to 30% sucrose (w/v) contained drinking water (HSD group) showed a significant rise in their body weights over the control mice (NCD) (Fig. 1*B*). In line with reports that mention excessive carbohydrate intake to induce liver fattiness (26), we found dense lipid droplets and vacuolar degeneration within the liver section of HSD mice, as compared with NCD mice post-H&E and oil red O staining, as depicted in the representative images (Fig. 1*C*). Furthermore, we also found transcript levels of hepatic DNL regulators (ACC, FASN, ChREBP) were increased in HSD exposed mice liver as compared with the NCD (Fig. 1*D*). As we had recently identified sorcin as a novel regulator of ChREBP-linked DNL (20), we assessed for sorcin level in the HSD liver tissue. We saw significant reductions in the transcript as well as the protein level of hepatic sorcin in HSD liver over NCD liver (Fig. 1, *E*–*G*), whereas sorcin levels remained unaltered in white adipose tissue (Fig. S1). So, as to understand the physiological mechanism underlying dietary carbohydrate–induced liver sorcin reduction and enhanced ChREBP-linked DNL, we generated a mouse model with adenoviral-mediated overexpression of sorcin (Fig. 1*H*). Following the adenoviral treatment regimes, Ad-SRI injected HSD mice showed a significant reduction in their body weights as compared with the control counterpart (Fig. 1*I*). Western blot analysis of hepatic tissue confirmed the significant expression of exogenous sorcin within the liver of Ad-SRI injected mice, as compared with respective control (HSD mice) (Fig. 1, *J* and *K*).

Next, we wanted to determine whether the reduced sorcin level in the liver of HSD mice associates with enhanced ChREBP nuclear protein abundance in liver. Immunoblotting of subcellular fractionated liver lysates revealed significant rise in the abundance of ChREBP protein in the nuclear lysate of HSD mice group as compared with NCD, and markedly, nuclear ChREBP levels decreased significantly after adenoviral overexpression of sorcin (Ad-SRI) in the HSD group (Fig. 1, *L* and *M*). These results suggested that ChREBP is translocated into the nucleus in response to high sucrose diet, whereas Ad-SRI reduced the sucrose-stimulated nuclear entry of ChREBP, which is required for its transactivation. Our *in vivo* observations proposed high sucrose diet to reduce hepatic sorcin levels and enable enhanced cytosol-nuclear shuttling of ChREBP, eventually causing hepatic dyslipidemia.

Further to delineate the underlying mechanism of high carbohydrate diet–induced enhanced ChREBP nuclear localization, we developed an *in vitro* setup, by treating the hepatic cell line (HepG2) with low (5 mM) and high (30 mM) glucose concentrations. Western blot analysis depicted high glucose to reduce sorcin protein levels in HepG2 cells (Figs. S2 and S3). To strengthen our observation of high glucose–mediated enhanced intracellular lipid accumulation is because of ChREBP’s transactivation *via* sorcin, we examined ChREBP subcellular localization, in response to low and high carbohydrate. Microscopic studies deciphered increased nuclear accumulation of ChREBP, in high glucose, as compared with low glucose (Fig. 1*N*). To further decipher the role of sorcin in high glucose–mediated ChREBP’s nuclear localization, we overexpressed m-Sorcin-FLAG in hepatocyte cells and performed ChREBP’s subcellular localization. Interestingly, sorcin overexpression in high glucose sequestered a major pool of ChREBP within the cytosol (Fig. 1N, O). The *in vitro* results thus reinforced the role of sorcin in cytosolic sequestration of ChREBP in hepatocytes. Altogether, these data clearly show that the downregulation of hepatic sorcin is the major factor that influences enhanced nuclear translocation of ChREBP in response to high carbohydrates intake.

### Pharmacological inhibition of NF-κB p65 attenuates high glucose–induced hepatic lipid accumulation

**Figure 2:**
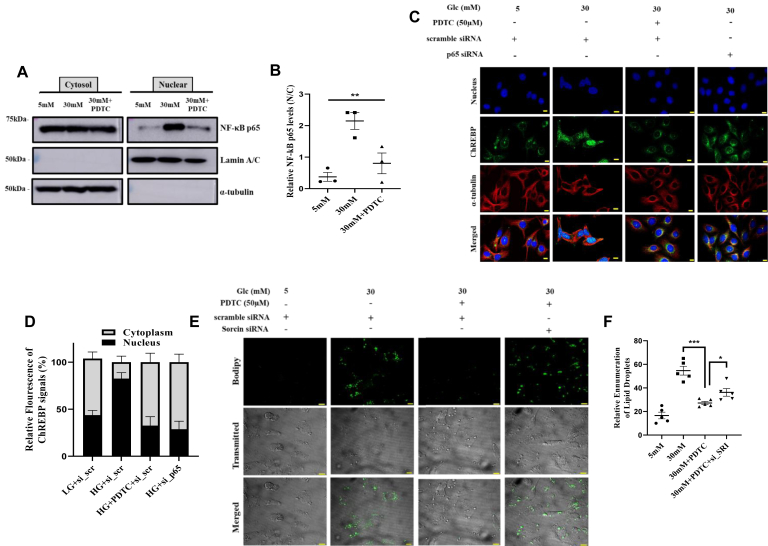
**Pharmacological inhibition of NF-κB p65 attenuates high glucose-induced hepatic lipid accumulation.***A* and *B*, qualitative and quantitative representation of NF-κB p65 in the subcellular fractionation lysates of cells treated with depicted conditions (*B*, mean ± SEM, ∗∗*p* < 0.005). *C* and *D*, immunofluorescence images of subcellular localization of ChREBP in presence PDTC or NF-κB p65 siRNA under low/high glucose conditions *via* immunocytochemistry in HepG2 cells (Image scale bar is 10 μm), with the quantification of ChREBP signals (nuclear: cytoplasmic ratio). *E* and *F*, representative microscopic images of Bodipy staining of HepG2 cells depicting abundance of intracellular lipid droplets in different conditions as depicted, inferring the extent of lipogenesis (Image scale bar is 10 μm) (*F*, mean ± SEM, ∗*p* < 0.05, ∗∗∗*p* < 0.0005). ChREBP, carbohydrate response element-binding protein; NF-κB, nuclear factor kappa-light chain enhancer of activated B cells; PDTC, pyrrolidine dithiocarbamate.

Our previous observations motivated us to delineate the mechanism behind the regulation of hepatic sorcin in response to carbohydrate treatment. Dietary components like high fat are reported to augment NF-κB p65 activation and induce lipogenesis in the liver (27, 28, 29). Thus, we aimed to study the effect of a high carbohydrate diet on NF-κB p65 activation. Posttranslational modifications of NF-κB p65 subunits, like phosphorylation of p65 subunit at serine536, activate NF-κB p65 and enables its nuclear entry (30). Phosphorylation of the p65 subunit is required for the optimal p65-mediated transactivation potential of NF-κB. Thus, we assessed the extent of phosphorylation-mediated nuclear translocation of NF-κB p65 in presence of low and high glucose and PDTC (an inhibitor of NF-κB). Surprisingly, we observed high glucose (30 mM) to enrich nuclear abundance of NF-κB p65 as compared with low glucose (5 mM), and also, PDTC was able to block high glucose induced translocation of NF-κB p65 (Fig. 2, *A* and *B*). We then questioned whether high glucose–induced activation of NF-κB p65 to have any link with subcellular movement of ChREBP in the similar nutritional stress. Interestingly, we did see high glucose–induced nuclear abundance of ChREBP to be significantly reduced in both pharmacological-induced NF-κB inhibition (PDTC) and NF-κB knockout state using siRNA for NF-κB p65 (Fig. 2, *C* and *D*). Knockdown was specific as reflected on immunoblot by approximately 90% reduction of NF-κB p65.

Additionally, we also assessed the phenotypic outcome of NF-κB p65 inhibition on ChREBP-induced hepatic intracellular lipid accumulation. High glucose (30 mM) treatment showed increased Bodipy-stained lipid droplets as compared with low glucose (5 mM). Pharmacological inhibition of NF-κB p65 abrogated high glucose–induced intracellular lipid droplet abundance. To further evaluate the participation of sorcin in fatty liver attenuating properties of NF-κB p65 inhibitor (PDTC), we knocked down sorcin using sorcin siRNAs in cultured HepG2 cells (Fig. S4) and later assessed the outcome through Bodipy staining. Interestingly, PDTC-induced reduction in intracellular lipid droplets was blunted in sorcin knocked down cells (Fig. 2, *E* and *F*). These observations recommended NF-κB p65 to have a pivotal role in high glucose–induced elevated hepatic lipid accumulation, and sorcin can be critical in the same axis.

### High glucose stimulates repression of hepatic sorcin *via* NF-κB p65-NCoR1 signaling

**Figure 3:**
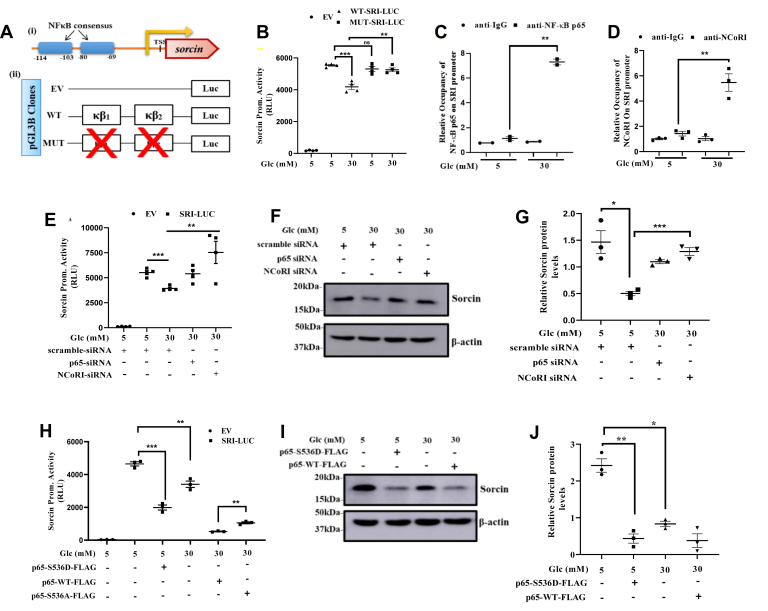
**High glucose stimulates hepatic *sorcin* repression *via* NFκB-p65-NCoRI signaling.***A*, (*i*) diagrammatic portrayal of sorcin promoter with both the NF-κB consensuses, as analyzed using EPD tool (Expasy), (*ii*) schematic representation of the sorcin promoter luciferase reporter constructs portraying the WT and mutant (MUT). *B*, relative light units of sorcin promoter activity in stipulated conditions, inferring high glucose–mediated sorcin repression to be dependent on NF-κB consensuses (mean ± SEM, ∗*p* < 0.05, ∗∗*p* < 0.005). *C* and *D*, qPCR levels of NF-κB consensuses on the chromatin elute post immunoprecipitation using NF-κB p65 and NCoRI antibodies, in contrast to IgG antibody. *E*, sorcin reporter assay under NF-κB p65 and NCoRI knockdown in presence of high glucose condition (mean ± SEM, ∗∗*p* < 0.005, ∗∗∗*p* < 0.0005). *F* and *G*, qualitative and quantitative levels of sorcin protein in presence of siRNA–mediated silenced p65 and NCoRI states along with mentioned glucose conditions, as depicted (mean ± SEM, ∗*p* < 0.05, ∗∗∗*p* < 0.0005). *H*, sorcin promoter activity in presence of transient overexpression of p65 constructs as depicted, inferring dependency of high glucose–mediated sorcin repression with S536 residue level phosphorylation of NF-κB p65 (mean ± SEM, ∗∗*p* < 0.005, ∗∗∗*p* < 0.0005). *I* and *J*, qualitative and quantitative representation of sorcin protein level in presence of transiently overexpressed p65 constructs and mentioned glucose conditions (mean ± SEM, ∗*p* < 0.05, ∗∗*p* < 0.005). ChREBP, carbohydrate response element-binding protein; NCoRI, nuclear receptor corepressor 1; NF-κB, nuclear factor kappa-light chain enhancer of activated B cells.

We next examined whether NF-κB p65 directly stimulates hepatic sorcin repression. Through bioinformatics tool, we came across NF-κB consensuses on human sorcin promoter (Fig. 3A-*i*). Hence, we prepared a luciferase reporter plasmid of human sorcin promoter with both the putative functional NF-κB consensuses found near (−69 to −80 bp) and (−103 to −114 bp) (Fig. 3A-*ii*). Following transfections and luciferase assay, we observed that cells exposed to high glucose showed reduced sorcin promoter activity in response to high glucose as long as two NF-κB sites were intact. Mutation of both the NF-κB sites abrogated the high glucose–mediated transcriptional repression of sorcin (Fig. 3*B*). Connecting with our previous observations, our results confirmed high glucose to stimulate nuclear localization of NF- κB p65followed by repressing sorcin promoter activity.

We, then next assessed the presence of activated NF-κB p65 (pNF-κB) on the sorcin promoter through ChIP assay. qPCR analysis of NF-κB specific consensus, from chromatin elutes obtained post immunoprecipitation with pNF-κB and IgG antibodies, showed high glucose treated cells to have greater interaction of NF-κB on sorcin promoter, as compared with low glucose (Fig. 3*C*). This observation seemed a little intriguing, as high carbohydrate diet–mediated NF-κB p65 activation had a negative co-relation with the hepatic sorcin abundance. Thus, we hypothesized presence of an additional transcriptional co-repressor. Following the cited literature (30), we assessed for the presence of nuclear receptor corepressor 1 (NCoRI), along with p65 protein on sorcin promoter. Interestingly, we did see enriched occupancy of NCoRI on NF-κB p65 consensus of sorcin promoter in chromatin elutes of high glucose exposed cells, as compared with the control (Fig. 3*D*). To further authenticate the association of NF-κB p65 or NCoRI with transcriptional repression of sorcin, we used siRNA specific of NF-κB p65 and NCoRI respectively and performed a sorcin reporter assay. Knockdown was specific as reflected on immunoblot by significant reduction of NF-κB p65 and NCoRI (Figs. S5 and S6). As depicted (Fig. 3*E*), on NF-κB p65 or NCoRI silencing, cells showed vivid perturbation in high glucose–induced reduction in sorcin promoter activity, respectively. Also, when assessed at the protein level from the total cell lysate, NF-κB p65 and NCoRI knockdown in high glucose conditions did significantly exhibit protection from high glucose–induced sorcin downregulation (Fig. 3, *F* and *G*).

To further validate our observation of NF-κB p65 mediating high glucose–stimulated sorcin downregulation, we used S536 mutants of p65-WT-FLAG construct and validated the role of NF-κB p65 (through S536) in carbohydrate stress–induced sorcin expression. Cells overexpressing p65-WT-FLAG construct significantly reduced sorcin promoter activity in presence of high glucose, whereas overexpression of the phospho-null (p65-S536A-FLAG) construct greatly attenuated the high glucose–induced repression of sorcin promoter activity. To our immense surprise, cells overexpressing the phospho-mimic (p65-S536D-FLAG) reduced sorcin promoter activity in presence of low glucose (Fig. 3*H*). This observation kindled an authentication of NF-κB p65 phosphorylation at S536 residue to be pivotal in high carbohydrate–mediated sorcin gene regulation. We also found a similar trend as observed in promoter activity when we assessed the transcript level endogenous sorcin in presence of these p65 constructs (Fig. S7). To further substantiate our observations, we transfected HepG2 cells with p65-S536D-FLAG and p65-WT-FLAG and maintained them in low and high glucose conditions and determined the sorcin protein levels. As shown in Figure 3, I and *J*, overexpression of p65-WT construct significantly reduced sorcin expression in presence of high glucose, whereas overexpression of the phospho-mimic greatly suppressed sorcin protein levels in presence of low glucose. Taken together, all our observations strongly highlight the crucial importance of NF-κB p65-NCoRI complex in mediating high glucose–induced repression of hepatic sorcin levels.

### Pharmacological inhibition of NF-κB p65 ameliorates high sucrose–induced fatty liver pathology *in vivo*

**Figure 4:**
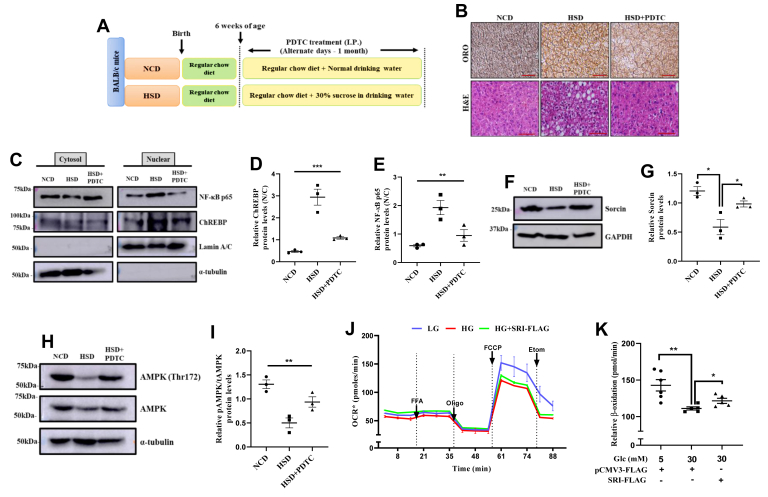
**Pharmacological inhibition of NF-κB p65 ameliorates high sucrose–induced fatty liver pathology *in vivo*.***A*, schematic description of the treatment regimens categorized in the *in vivo* study aiming NFκB inhibition through PDTC. *B*, histological analysis of liver tissue sections through oil red O staining and H&E staining from all the mice groups portraying the extent of intracellular accumulated lipid droplets (Image scale bar is 500 μm). *C*–*E*, qualitative and quantitative representation of ChREBP (*D*) and NF-κB p65 (*E*) levels in subcellular fractionated liver lysates, normalized to loading control (mean ± SEM, ∗∗*p* < 0.005, ∗∗∗*p* < 0.0005). *F* and *G*, qualitative and quantitative representation of sorcin abundance in the liver lysates of respective mice groups of our *in vivo* study (mean ± SEM, ∗*p* < 0.05). *H* and *I*, qualitative and quantitative representation of phosphorylated AMPK (Thr172) levels in the liver lysates of respective mice groups (mean ± SEM, ∗∗*p* < 0.005). *J* and *K*, mitochondrial respiration potential emphasizing ability to oxidize exogenous lipid was assessed in HepG2 cells exposed to differential glucose concentrations through Seahorse flux analyser (mean ± SEM, ∗*p* < 0.05, ∗∗*p* < 0.005). AMPK, AMP-activated protein kinase; ChREBP, carbohydrate response element-binding protein; NF-κB, nuclear factor kappa-light chain enhancer of activated B cells; PDTC, pyrrolidine dithiocarbamate.

To evaluate whether NF-κB p65 participates in regulating sucrose-mediated transactivation of ChREBP and thereby modulate hepatic lipid accumulation in *in vivo*, we administered PDTC through I.P. injections to the high sucrose-fed mice, with respective control groups (Fig. 4*A*). As observed in our *in vitro* setup, hepatic lipid attenuating properties of PDTC was significantly vivid in our *in vivo* model too, as both oil red O staining and H&E staining of liver sections (Fig. 4*B*). Next, we examined NF-κB p65 subcellular localization in response to high sucrose diet and effect of PDTC treatment on the same. Interestingly, we found increased NF-κB p65 nuclear localization in HSD exposed mice, which was prevented by PDTC. Overall, PDTC treatment did curtail nuclear abundance of NF-κB, as observed otherwise in HSD exposed mice (Fig. 4, *C* and *D*). Moreover, PDTC treatment also showed slight reduction in the nuclear levels of ChREBP in HSD background (Fig. 4, *C* and *D*), indicating PDTC to effectively block high sucrose–induced hepatic lipid accumulation by restricting nuclear localization of ChREBP, which is otherwise required for optimal sucrose-mediated enhanced DNL. To examine the effect of high sucrose diet and/or PDTC on sorcin, we measured sorcin protein expression profile in NCD and sucrose-fed mice (HSD) liver in presence and absence of PDTC treatment (Fig. 4, *F* and *G*). Interestingly, we observed that in HSD-saline mice group resulted in significant repression of sorcin levels in the liver, whereas PDTC treatment protects sorcin from such downregulation (Fig. 4, *F* and *G*). Taken together, these results indicate PDTC to specifically block high carbohydrate diet–stimulated nuclear localization of NF-κB p65, which is required for optimal p65-mediated transactivation potential of NF-κB to suppress sorcin expression which in turn enhances nuclear localization of ChREBP.

To further dissect the antilipogenic mechanistic axis of PDTC, on hepatic lipid homeostasis, through another potent energy metabolism energy sensor protein, AMP-activated protein kinase (AMPK) activation is expected to coordinate the partitioning of fatty acids between oxidative and biosynthetic pathways by increasing fatty acid oxidation (FAO) capacity and inhibiting DNL, respectively. We thus measured the phosphorylation of AMPK at Thr172, as a surrogate for AMPK activation, in the liver lysates of our experimental mice group. Interestingly, we found that high sucrose (HSD liver lysates) exposure blunted AMPK(Thr172) phosphorylation, whereas PDTC restrained the downregulation of phosphorylation of AMPK (Thr172) led by high sucrose, inferring PDTC to also signal through AMPK to perturb hepatic dyslipidemia (Fig. 4*H*). Thus parallelly, our data sets also hint NF-κB p65 inhibition to induce AMPK activation and attempt a broader protection against hepatic triglyceride accumulation in mice fed a high-sucrose diet.

High carbohydrate intake induced hepatic dyslipidemia is an outcome of enhanced DNL along with collateral signaling cascades like increased fatty acid uptake or reduced lipolysis or lipid export from the liver (31). As lipogenesis is preferentially coupled with lipolysis for lipid homeostasis, the lipid oxidation potential of cells in the presence of high glucose concentrations and in presence of sorcin overexpression. For this, we analyzed the oxygen consumption rate (OCR) in the presence of an exogenous fatty acid mixture using the Seahorse Flux Analyser approach. Basal respiration and ATP synthase–dependent OCR changes did not seem to have significant differences in presence of 5 mM and 30 mM glucose. But interestingly, 30 mM glucose treated cells did show significant reduction in the 2-[2-[4-(trifluoromethoxy)phenyl]hydrazinylidene]-propanedinitrile-induced mitochondrial quest to oxidize the exogenously provided fatty acid mixture as compared with the 5 mM glucose control, inferring perturbed lipolytic axis in presence of 30 mM glucose exposure. Also, to our great surprise, sorcin overexpression in 30 mM glucose treated cells showed a remarkable reversal of mitochondrial potential to oxidize exogenous lipid (Fig. 4, *J* and *K*). All the above attributes observed in the almost absence of sorcin (*i.e.*, in carbohydrate treatment) and its overexpression conditions, vividly present sorcin to be a novel hub protein dictating intracellular hepatic lipid partitioning. Collectively, our data sets confirm maintaining of hepatic sorcin level in high carbohydrate treatment to significantly reverse high carbohydrate–induced fatty liver pathologies, by blocking ChREBP driven hepatic DNL and also elevating the lipid oxidation axis.

## Discussion

Incidences of metabolic dysfunction are elevating at an alarming rate across the globe. NAFLD characterized by excessive hepatic fat accumulation is a multifactorial clinical manifestation, without a history of alcohol abuse. NAFLD invokes serious concerns owing to no treatment modalities or no “magic bullet” which could pharmacologically target its occurrence or rescue (32). It also becomes trivial to dissect this pathophysiology as regulation of hepatic lipogenesis involves the interplay of various transcription factors and/or nuclear receptors, varying hormonal stimuli, nutrients, and/or environmental toxicants (20, 33). Excessive intake of carbohydrates, as well as high-fat meals, is reported to be a major risk factor of NAFLD development (34). Because excessive circulating sugar can force an anabolic flux within the liver worth fatality, it becomes imperative to understand sugar responsive pathways within the liver for therapeutic advances.

ChREBP is a glucose-sensitive lipogenic transcription factor majorly found in lipid metabolizing organs like adipose tissue and liver. ChREBP-driven DNL within hepatocytes is recorded to be one of the master regulators of NAFLD (35). Structural studies of ChREBP present several posttranslational modifications to unbridle ChREBP from the cytosolic sequestration fostering nuclear entry. Although there are kinases that phosphorylate ChREBP and limit it within the cytoplasm, we through our study highlight an adaptor protein sorcin to regulate nucleo-cytosolic ChREBP trafficking. Sorcin is a calcium sensor protein, belonging to the penta-EF-hand family is a highly conserved protein among mammals (36). It is reported to interact and localize ChREBP within the cytoplasm of hepatocytes and pancreatic β-cells (20, 21). In our study, we observed high carbohydrate diet to reduce both mRNA and protein levels of hepatic sorcin. This phenomenon enriched nuclear entry of ChREBP, prompting transactivation of ChREBP-dependent genes like FASN, ACC. The upregulation of these genes enforced hepatic dyslipidemia, thereby provoking the graduation of fatty liver pathologies. Interestingly, exogenous sorcin blocked high carbohydrate–induced ChREBP hypertransactivation and thereby attenuated lipogenesis, we also observe sorcin overexpression to accelerate lipid oxidation. Our observation presented exogenous sorcin to rescue high carbohydrate diet–compromised mitochondrial potential toward enhanced β-oxidation. High carbohydrate diet–induced oxidative stress would be an early priming agent damaging mitochondria, which later reduces the quest of hepatic mitochondria to oxidize exogenous lipid (37, 38). Overexpression of sorcin in high glucose condition seemed to retrieve the health of mitochondria (as observed by the baseline OCR, Fig. 4*K*) and thereby enforced FAO in hepatocytes. Thus, through our work, we propose hepatic sorcin to have a plausible regulator of hepatic lipid homeostasis.

Although sorcin is a well-studied protein in the field of calcium cycling, intracellular calcium homeostasis, multidrug resistance, cancer, etc., there exists no article that mentions any signaling axis for its regulation. As NF-κB is one of the major transcription factors that regulate development, inflammatory responses, and tackle nutritional stress (high carbohydrate diet/high-fat diet) by fostering lipogenic stimulus (39, 40, 41), we were keen to assess high carbohydrate–induced effect on NF-κB signaling. Subcellular fractionation (liver tissue) and ChIP assay demonstrated a high carbohydrate diet to phosphorylate p65 at S536 and thereby activate its transcriptional activity. Phosphorylation of p65 at S536 residue enforced interaction with NcoRI and thereby repressed transcription of sorcin, following the cited studies (42, 43). As our focus was to understand the importance of sorcin in regulating high carbohydrate diet–induced ChREBP-driven hepatic steatosis, it seemed imperative to understand the mechanism behind sorcin downregulation. Thus, through this study, we for the first-time present NF-κB p65 to mediate the repression of hepatic sorcin and in turn modulate dietary carbohydrate induced DNL. Thus, along with other cited reports emphasizing NF-κB p65 in cases of fatty liver pathologies (29, 44, 45), we propose NF-κB–Sorcin–ChREBP be a potential axis worth therapeutic explorations.

Our current observations prompted us to explore the probable signaling cascade which induces high carbohydrate diet–mediated NF-κB p65 activation. IKK1/2 stands out to be a probable kinase that transduces high carbohydrate diet–induced p65 activation, as high glucose is observed to regulate its expression and activity. Another study, in accordance, highlights the O-GlcNacylation of IKK to enhance p65 activation by phosphorylating it at S536 (27, 46, 47, 48, 49, 50). At this juncture, we speculate HSD to induce the O-GlcNacylation of IKK and thereby enhance NF-κB activity, a possibility that remains to be tested. Another interesting aspect that appears to stand out includes no aggravation (absence/repressed) of an inflammation and insulin resistance, in our study, as NF-κB p65 activation induces an inflammatory response and thereby exacerbates insulin sensitivity. This could have been owing to multiple reasons like NF-κB p65 is studied to portray phosphorylation residue-specific remodels gene-specific transcriptional regulation (24) or ChREBP–dependent production of lipid intermediates which can act as an anti-inflammatory and insulin-sensitizing lipid mediators into systemic circulation acting through *cell-autonomous or/and nonautonomous manner* (1, 34, 51, 52, 53, 54, 55).

Given that the growing population is at risk of many pathological conditions linked with fatty liver, it stands relevant to identify molecular mechanisms through which diet-induced pathogenesis of fatty liver progresses. Our data suggest a critical role of NF-κB p65 in regulating ChREBP’s nuclear localization to increase hepatic lipid accumulation.

Based on these findings, we propose that the NF-κB p65–Sorcin–ChREBP cytosolic complex to be a potential site for pharmacological interventions as an effective therapy for reducing high carbohydrate diet–induced fatty liver disease.

## Experimental procedures

### *In vivo* studies

Animal studies were approved by the Jamia Hamdard Animal Use and Care Committee and were performed in 6- to 8-week-old BALB/c male mice. The mice were maintained on regular chow diet and housed at a temperature of 25 °C with 12-h light/dark cycles. Ten mice were divided into two major groups, wherein the untreated control mice (were kept on normal drinking water, whereas the test group (HSD) was fed with 30% sucrose (w/v) through drinking water *ad libitum*). Three mice/group (mice were selected on the basis of blood glucose, serum ALT, AST, TG) were used for transcripts levels quantification in liver tissue. For PDTC experiments, we administered 1 mg/kg body weight of PDTC through I.P. injections to the high sucrose fed mice, with respective control groups. All the experiments were performed following guidelines stated and approved by the Animal Ethics Committee of Jamia Hamdard. Adenoviral overexpression of exogenous human sorcin in the *in vivo* model was carried out as described previously (20).

### Preparation of recombinant SRI-adenovirus

Sorcin adenoviral construct (Ad5-SRI) was prepared following the Gateway cloning methodology (Thermo). h-SRI cDNA was sequentially cloned into pAD/DEST vector, after d-TOPO vector. SRI cloned pAD vector was then linearized using PacI and then transfected into HEK293A cells for amplification using lipofectamine 2000 (Invitrogen). High titer stocks of amplified recombinant adenovirus were purified using PureVirusTM Adenovirus Purification Kit (CELL BIOLABS INC) as per the protocol. Viral titers were determined, diluted in 0.9% saline and administered approximately 10^9^ pfu/mice through tail vein injection. All respective control mice were injected with p-Ad-empty (no insert) for maintaining the exact similar vehicle control.

### Gene expression level studies

Cells/tissues were lysed using TRI-reagent, and total RNA was extracted. 1 μg of total RNA was processed for cDNA as per the manufacturer’s guidelines (iScript cDNA synthesis kit, *Bio-Rad*). Quantitative PCR was then performed using the SYBR mix following iQ SYBR Green Supermix, *Bio-Rad*. Gene expression analysis was carried out using 2^−ΔΔCT^ method and normalized with 18srRNA, HPRT gene expression.

### *In vitro* experiments

HepG2 cells were used for several *in vitro* studies. To mimic high carbohydrate condition in *in vitro* model, cells were treated with 5 mM and 30 mM glucose solutions, for a stipulated period, specified experimentally. Transfection experiments were performed using Lipofectamine 3000 (*Thermo*) or Lipofectamine RNAimax (*Thermo*) for plasmid or siRNA respectively, strictly following the manufacturer's protocol. siRNA sequences used in the study is enlisted in the supplementary file (Table S1). For, performing site-directed mutation’s, Q5-SDM kit (*NEB*) was used, and the primers were prepared using the *NEBase changer* link. The mutants were successfully sequenced using respective plasmid specific sequencing primers then used for *in vitro* experiments.

### Nuclear localization analysis of ChREBP

ChREBP-driven lipogenic upregulation expects cytoplasmic-nuclear shuttling. This localization was tracked using immunocytochemistry (ICC) and subcellular fractionation following the protocol described previously (20). ICC was performed in HepG2 cells using 1:100 diluted ChREBP and 1:200 diluted anti-rabbit. Microscopic images were captured using a *Zeiss* fluorescence microscope. ICC experiments were repeated three times, and the best representative images have been represented. Subcellular fractionation was majorly performed from liver tissues using NE-PER nuclear and cytoplasmic extraction kit (*Thermo*), and an equal concentration of nuclear pools were loaded for Western blot, respectively.

### Lipid droplet staining

DNL–induced intracellular lipid droplets were qualitatively studied using Bodipy staining (*in vitro*) and Oil Red O (tissue). For HepG2 cells, cells were stimulated with 5 mM and 30 mM Glc for 48 h and then stained with Bodipy. Briefly, cells were washed 1X-PBS and fixed with 3% PFA for 10 min at RT. Following two PBS washes, cells were stained with Bodipy (2 μM) for 30 min at 37 °C. Cells were mounted using mounting media (*Sigma*), following three PBS washes and later visualized on a confocal microscope (A1+, *Nikon*), and representative images have been presented. Liver tissues were embedded in Tissue-Tek, frozen sections were then processed for oil red O staining using a routine method (20). Images were captured using *Nikon Eclipse TS* 100 confocal microscope.

### Chromatin immunoprecipitation and sorcin promoter studies

S536 phosphorylation of p65 enforces its nuclear entry and thereby regulates transcriptional processes. Sorcin promoter was analyzed using the EPD tool accessed from ExPASy. Significant interaction sites of NF-κB were found on the promoter at 75 base pairs upstream to the transcription start site. Chromatin immunoprecipitation to assess occupancy of phosphorylated p65 along with co-repressor on sorcin promoter was performed as described previously (20). Primers enriching the domain containing NF-κB consensus (−313 bps to −22 bps) were designed using the EPD tool.

Sorcin promoter reporter construct was prepared by cloning NF-κB consensus containing a fragment of human sorcin promoter(from human gDNA) into the pGL3B construct (*Addgene*). To validate our hypothesis, we also mutated the same stretch of the promoter sequence by deleting both the two NF-κB consensuses (GGRRNNYYCC) site found near the following sites (−69 to −80 bp) and (−103 to −114 bp) and performed promoter activity. Primer sequences is shared in the supplementary file (Table S2).

### Western blotting

Tissues and cells were lysed using RIPA buffer mixed with protease inhibitor cocktail and phosphatase inhibitor (*Thermo*). A constant amount of protein was electrophoretically run on SDS gels and then transferred onto a 0.22μ nitrocellulose membrane (*BioRad*) at a constant voltage. Blots were probed with respective antibodies and then developed using ECL (*GE*) as per guidelines. Antibodies used in the study are enlisted in the supplementary file (Table S3).

### Exogenous fatty acids oxidation

Mitochondrial ability to undergo lipid oxidation was assessed in *in vitro* using Xp-Seahorse flux analyzer. Ten thousand cells were seeded in each well and later treated with 5 mM and 30 mM glucose concentrations for 48 h, respectively. Later, the cells were incubated with substrate limited media for 24 h, followed by FAO buffer, as suggested in the manufacturer’s protocol. Basal respiration was assessed followed with an injection of 1 mM free fatty acid mixture comprising of palmitate and oleate in ratio 2:1 into the wells to analyze the exogenous free FAO. Free fatty acid was then followed with oligomycin (2 μM), 2-[2-[4-(trifluoromethoxy)phenyl]hydrazinylidene]-propanedinitrile (1.5 μM), and Etomoxir (40 μM). As we had performed this study in the XFp system, we have presented the OCR (pmol/min) post normalization from all the studies.

### Statistical analysis

Most of the figures presented are as mean ± SEM of a minimum of three experiments unless mentioned. An unpaired student *t* test (for two groups) and one-way ANOVA test (for three groups) were used for all the statistical analysis purposes. For all the photomicrographs, the scale usually set was 10 μM, unless specified.

## Data availability

The data sets supporting the conclusions of this article are included within the article and its supporting information.

## Supporting information

This article contains supporting information.

## Conflict of interest

No potential conflicts of interest relevant to this article were reported.
