# Supplementary material for: NF-κB p65 regulates hepatic lipogenesis by promoting nuclear entry of ChREBP in response to a high carbohydrate diet
Source: J Biol Chem. 2021 Apr 27;296:100714. doi: 10.1016/j.jbc.2021.100714 (PMC8144664; doi:10.1016/j.jbc.2021.100714)
Supplement: Supplemental Figures S1–S8 and Tables S1–S3 [file mmc1.docx]

**NF-κB** **p65 regulates hepatic lipogenesis by promoting the nuclear entry of ChREBP in response to high carbohydrate diet**

**P. Vineeth Daniel^$^, Surbhi Dogra^$^, Priya Rawat^$^, Abhinav Choubey^$^, Aiysha Siddiq Khan^‡^, Sangam Rajak**^¥^**, Mohan Kamthan^‡^*, Prosenjit Mondal^$^***

***^$^*** School of Basic Sciences, Indian Institute of Technology Mandi, Mandi-175001, H.P, India,

*^¥^* Department of Endocrinology, Sanjay Gandhi Post Graduate Institute of Medical Sciences, Lucknow

***^‡^*** Department of Biochemistry, School of Chemical and Life Sciences Jamia Hamdard, New Delhi, India

***** To whom correspondence should be addressed:

**Mohan Kamthan**, PhD

Department of Biochemistry,

School of Chemical and Life Sciences Jamia Hamdard,

New Delhi, India

Email: kamthan.mohan@gmail.com

Phone no: 91-8127504219

**Prosenjit Mondal**, PhD.,

School of Basic Sciences,

Indian Institute of Technology

Mandi, H.P -175005, India

Email: [prosenjit@iitmandi.ac.in](mailto:prosenjit@iitmandi.ac.in).

Phone no: (91)1950267262

**Running Title: NF-κB regulates ChREBP through Sorcin in response to high carbohydrate diet.**

**Keywords:** ChREBP, Sorcin, NF-κB (p65), High carbohydrate diet, Dyslipidemia, Lipid Partitioning

- Table S1: **Sequence of siRNA used in the study**:

| **S.No.** | **siRNA** | **Sequence** | |
| --- | --- | --- | --- |
|  | | Sense | Antisense |
| 1. | ChREBP (s27386) | UGGUAUAUCCAGUAUGUGAtt | UCACAUACUGGAUAUACCAgg |
| 2. | Sorcin  (s13421) | GGCUUAUGGUUUCAAUGCUtt | AGCAUUGAAACCAUAAGCCgg |
|  | Sorcin  (s13422) | GCUGUGAAUUCAAUUGCAAtt | UUGCAAUUGAAUUCACAGCct |
| 3. | RELA (p65)  (s11914) | CCCUUUACGUCAUCCCUGAtt | UCAGGGAUGACGUAAAGGGat |
| 4. | NCoRI  (s201) | GGCUUAUGGAGGACCCUAUtt | AUAGGGUCCUCCAUAAGCCca |

- Table S2: **List of primer pairs sequences:**

| **Primers** | **Sequences for forward and reverse primers (5′ to 3′)** |
| --- | --- |
| hSorcin – Cloning-F | CACCATGGCGTACCCGGGG |
| hSorcin– Cloning-R | TTAAACACTCATGACACATTGAATGA |
| hChREBP F | AGAGACAAGATCCGCCTGAA |
| hChREBP R | CTTCCAGTAGTTCCCTCCA |
| hFAS F | CGGTACGCGACGGCTGCCTG |
| hFAS R | GCTGCTCCACGAACTCAAACACCG |
| hACC F | TGATGTCAATCTCCCCGCAGC |
| hACC R | TTGCTTCTTCTCTGTTTTCTCCCC |
| hSREBP-1c F | TCAGCGAGGCGGCTTTGGAGCAG |
| hSREBP-1c R | CATGTCTTCGATGTCGGTCAG |
| hβ-actin F | CCAACCGCGAGAAGATGACC |
| hβ-actin R | GGAGTCCATCACGATGCCAG |
| mSorcin F | GCACCAGCGGGAAGATCA |
| mSorcin R | TGAGAGCCCTCAGTTTGACACA |
| mACC-F | TGACAGACTGATCGCAGAGAAAG |
| mACC-R | TGGAGAGCCCCACACACA |
| mFAS-F | GCTGCGGAAACTTCAGAAAAT |
| mFAS-R | AGAGACGTGTCACTCCTGGACTT |
| mChREBP-F | ACTCGGACTCGGACACAGAC |
| mChREBP-R | AGGCTCAAGCACTCGAAGAG |
| m18S-F | GCAATTATTCCCCATGAACG |
| m18S-R | GGCCTCACTAAACCATCCAA |
| m-HPRT-F | TCCTCATGGACTGATTATGGACA |
| m-HPRT-R | TAATCCAGCAGGTCAGCAAAGA |
| ChIP- NF-κB -FP | GGGGTGGTGTATTGGTGTTGG |
| ChIP- NF-κB -RP | TGTGCGCCAGGCCTCT |

| **S.No.** | **Antibody** | **Company** | **Catalogue** |
| --- | --- | --- | --- |
| 1 | ChREBP | Novus Bio | NB400-135 |
| 2 | Sorcin | Thermo (Invitrogen) | PA5-23143 |
| 3 | β-actin | Cell Signalling Technology | 4970 |
| 4 | α-tubulin | Thermo (Invitrogen) | DM1A-62204 |
| 5 | GAPDH | Cell Signalling Technology | 5174 |
| 6 | Laminin A/C | Santacruz Biotech | sc-7293(346) |
| 7 | p-NF-κB (S536) | Cell Signalling Technology | 3033 |
| 8 | NCoRI | Cell Signalling Technology | 34271 |
| 9 | Anti-FLAG | Sigma | F1804 |
| 10 | Alexa- 488-anti-rabbit IgG | Jackson ImmunoResearch | 711-095-152 |
| 11 | Alexa- 647-anti-mouse IgG | Jackson ImmunoResearch | 115-605-006 |

- Table S3: **List of antibodies**:
- **Supplementary figures:**


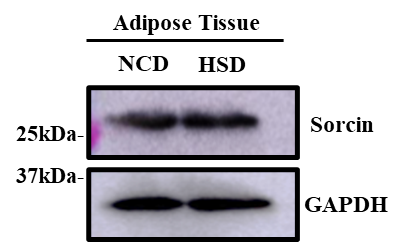


**S1**

**S2**


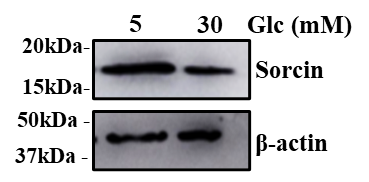


**S4**


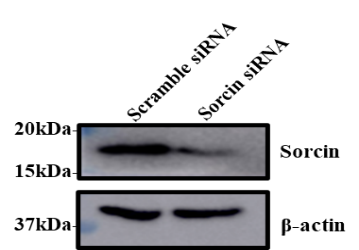


**S3**

**S7**


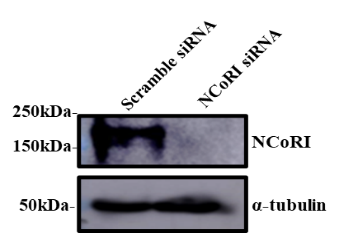


**S6**


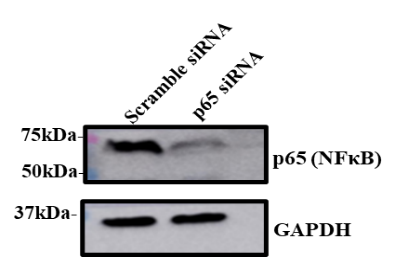


**S5**

**Supplementary Figure Legends:**

**S1.** Qualitative representation of sorcin protein abundance in adipose tissue from respective mice groups. **S2,S3.** Western Blot of Sorcin protein in low (5mM) glucose and high (30mM) glucose concentrations, from hepatic cell line lysate, with its respective densitometric analysis. **S4-S6.** Western Blot images inferring significant knockdown efficiency of respective proteins post their siRNA mediated silencing, as compared to the scramble controls, normalized to respective loading controls. **S7.** Relative sorcin transcript levels in presence of transiently overexpressed p65 constructs (as depicted) in the background of mentioned glucose conditions (mean±SEM, *p<0.05, **p<0.005).
